# Artificial intelligence in non-small cell lung cancer: transforming diagnosis, treatment, and prognostic evaluation

**DOI:** 10.3389/fmed.2026.1906478

**Published:** 2026-09-07

**Authors:** Shuaiyu Zheng, Chi Lv, Zhenyu Yao

**Affiliations:** 1 The First Affiliated Hospital, College of Clinical Medicine, Henan University of Science and Technology, Luoyang, Henan, China; 2 The First Affiliated Hospital of Zhengzhou University, Zhengzhou, Henan, China; 3 Clinical Medicine Research Centre, Sanya Hospital of Traditional Chinese Medicine (Hainan Hospital, Guangzhou University of Chinese Medicine), Sanya, Hainan, China

**Keywords:** artificial intelligence, digital pathology, NSCLC, personalized medicine, radiomics

## Abstract

Non-small cell lung cancer (NSCLC) is characterized by profound biological heterogeneity, frequently leading to late-stage diagnosis, recurrence, or metastasis. Conventional clinical pathways heavily depend on subjective visual interpretations, invasive tissue biopsies, and experience-based medical models that constrain individualized precision medicine. Identifying noninvasive, cross-scale tools for whole-disease-course assessment is critical for optimizing clinical decision-making and survival outcomes. This review critically synthesizes recent research progress and core algorithmic applications of artificial intelligence (AI) across the diagnostic, therapeutic, and prognostic continuum of NSCLC. In diagnostics, AI models enable rapid morphological screening and tumor subtyping on digital pathology slides, whereas multimodal imaging radiomics extracts microscopic features from macroscopic CT, PET/CT, and MRI scans to noninvasively identify tumor heterogeneity and map metastatic risk. Therapeutically, machine learning and large language models predict treatment responses, characterize tumor microenvironment dynamics, optimize perioperative surgical strategies, and accelerate drug discovery by screening chemical databases and decoding candidate drug mechanisms. Prognostically, multiscale fusion models integrate liquid biopsies, pathomics, radiomics, and clinical records to provide precise risk stratification for recurrence and survival outcomes. While AI has demonstrated transformative potential across the entire clinical lifecycle of NSCLC, significant technical bottlenecks remain, including severe data heterogeneity across healthcare institutions, the lack of clinical transparency in end-to-end “black box” architectures, and low-throughput biological validation. Future research must focus on standardizing data protocols, improving explainable AI, and incorporating multidimensional time series data to realize true intelligent precision medicine.

## 1 Introduction

Non-small cell lung cancer (NSCLC), the predominant histological type of lung cancer, accounts for approximately 85% of all lung cancer cases (1). Owing to the high biological heterogeneity of NSCLC, patients are often diagnosed at an advanced stage or face a persistently high risk of recurrence and metastasis even after undergoing radical resection, neoadjuvant chemotherapy, targeted therapy, and immunotherapy (2, 3). The conventional clinical pathway relies heavily on empirical visual interpretation by radiologists, invasive tissue biopsy, and experience-based medical models anchored in TNM staging (4). However, this paradigm is not only limited by subjective interpretation bias, high rates of missed diagnoses, and the inability to perform dynamic monitoring but also struggles to deeply mine the profound heterogeneity features driving tumor progression from macroscopic imaging and microscopic pathology, thereby constraining the advancement of individualized precision medicine in clinical practice (5–9). Therefore, identifying an efficient, noninvasive, and cross-scale tool for whole-disease-course assessment is critical for optimizing clinical decision-making and improving survival outcomes in patients with NSCLC.

Concurrently, artificial intelligence (AI) technologies, particularly deep learning and machine learning, are rapidly reshaping medical research and clinical practice (10). Supported by robust computational power and advanced algorithmic architectures, AI techniques have achieved breakthroughs in medical image processing, natural language processing, and multiomics big data mining (11). At the level of computer vision, deep learning models such as convolutional neural networks (CNNs), self-supervised learning, and neural architecture search (NAS) can automatically achieve high-precision three-dimensional segmentation of lesions and extract hundreds to thousands of digital features, both microscopic and macroscopic, that transcend the limits of human visual perception from small-sample or weakly supervised data (12, 13). In multiomics and text processing, ensemble learning methods, including XGBoost and random forest, and large language models can seamlessly integrate blood genomics, dynamic liquid biopsy sequences, and unstructured clinical semantics from electronic health records (EHRs) (14, 15). More importantly, the introduction of explainable AI (XAI) techniques, such as local interpretable model-agnostic explanations (LIME) and Shapley Additive exPlanations (SHAP), has progressively broken the “black box” nature of traditional deep learning models, enabling algorithms to output feature weights with clinical transparency and biologically causal orientation (16, 17). This accumulation of technological advances has laid a solid algorithmic foundation for the deep immersion of AI in the full lifecycle management of cancer.

In this review, we critically synthesize progress in the key algorithmic applications of AI across the diagnosis, treatment, prognosis, and whole-course disease management of NSCLC. This review adds value by organizing the evidence along the clinical continuum and by comparing validation design, generalizability, interpretability, and translational readiness across pathology, radiology, treatment, and prognostic modeling. This cross-scale perspective highlights where apparently strong model performance remains disconnected from reproducible clinical benefit. We focus on how AI supports the entire diagnostic and therapeutic continuum by systematically examining multiple dimensions, including microscopic ecosystem dissection, macroscopic phenotype-based noninvasive mining, and personalized whole-course disease management. Moreover, this review will delve into the current technical bottlenecks and clinical translational barriers faced by these studies and prospectively look forward to future AI-driven trends in lung cancer diagnosis and treatment, aiming to provide robust theoretical support for the design of future prospective clinical trials and the construction of digital precision diagnosis and treatment systems.

## 2 Application of AI technologies in the diagnosis of NSCLC

**Figure 1:**
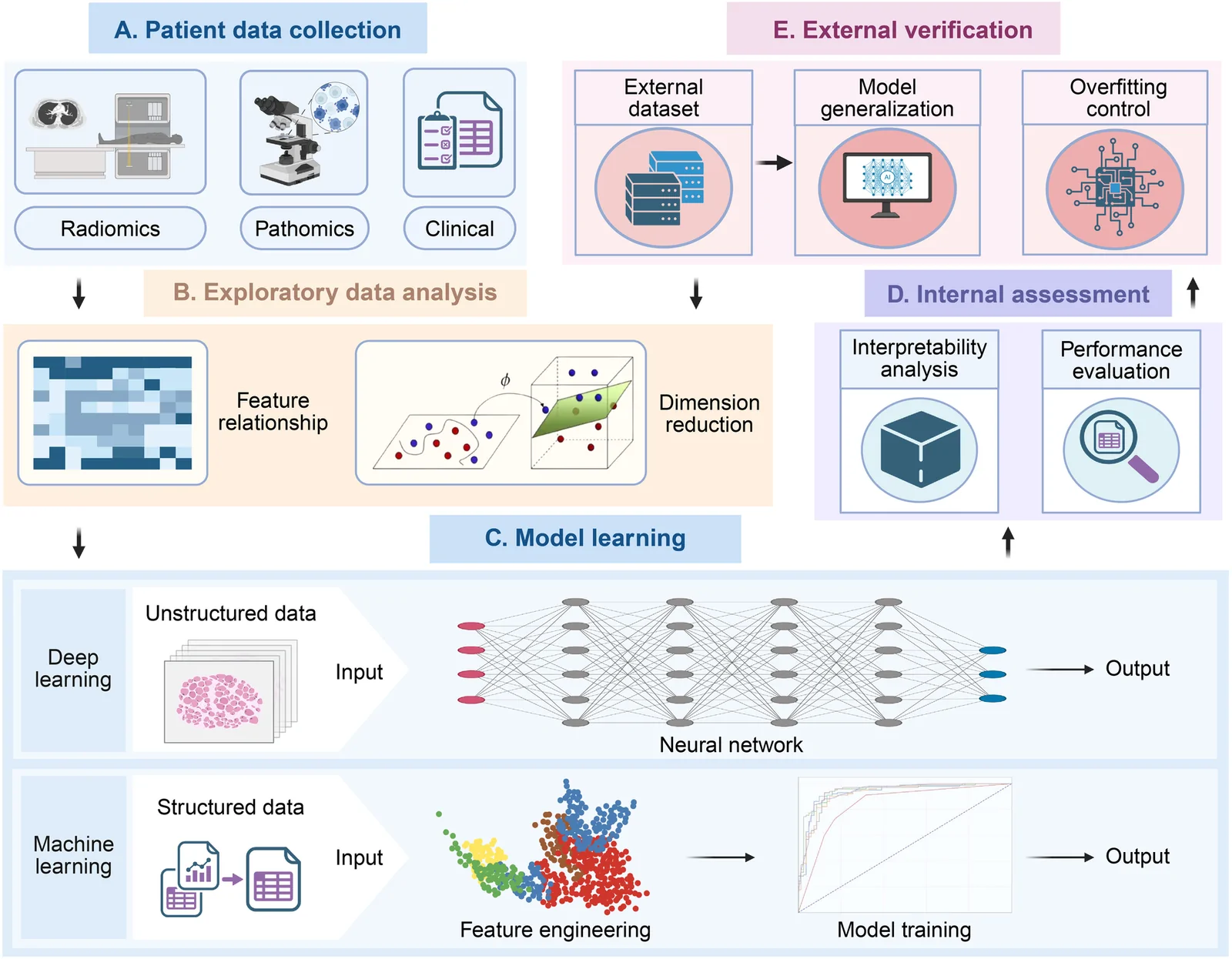
The basic process of AI-assisted diagnosis in NSCLC. **(A)** Data collection. **(B)** Exploratory data analysis. Explore the relationships among features and conduct feature engineering. **(C)** Model training. **(D)** Internal assessment. **(E)** external verification.

Accurate diagnosis and molecular phenotyping are fundamental to individualized treatment in NSCLC. However, traditional diagnostic pathways are frequently limited by subjective interpretations and invasive procedures. In this section, we review the transformative applications of artificial intelligence in NSCLC diagnosis, specifically focusing on microscopic AI-assisted histopathological evaluation and macroscopic noninvasive multimodal imaging radiomics for precise tumor subtyping and genetic prediction (Table 1) (Figure 1).

**Table 1:** Application of AI technologies in the diagnosis of NSCLC.

Imaging modality	Model	Classifier (ML)	Design/source	Sample/valid	Feature/valid	Purpose and results	Author and year	Reference
Histopathological images	xDL	CNN	Public database	15,000 lung images	Grad-CAM and Occlusion Sensitivity	classification between healthy tissue, adenocarcinoma, and squamous cell carcinoma, 2-class (Benign vs. Malignant): Accuracy: 99.69%, AUC: 99.75%	Civit-Masot et al., 2022	(23)
Autofluorescence imaging	xDL and GAN	CNN/DNN	TMA cores from patients	631 TMA cores	Grad-CAM and t-SNE	Label-free pathological subtyping of NSCLC and clinical-grade virtual IHC staining,Binary-class AUC: Up to 0.981 (Intensity) and 0.998 (FLIM)	Zang et al., 2026	(24)
Histopathological images	DL	CNN	Multi-source	21,533 WSIs	ROI and GNN	Direct identification of ALK and ROS1 fusions in NSCLC from H&E-stained slides, Sensitivity 100%, Specificity 100%, AUC = 1.00	Mayer et al., 2022	(26)
Histopathological images	DL	CanvOI 1.1 + MIL Ensemble	Multi-center and International Cohorts	Train/Dev: >4,425 WSIs, External Test: 968 WSIs	Unsupervised foundation embeddings	Directly identify 4 actionable NSCLC biomarkers from routine H&E slides. ALK: 0.96 BRAF V600E: 0.88 EGFR: 0.87 MET ex14: 0.83	Rolfo et al., 2026	(27)
Histopathological images	ML/DL	HALO® AI DenseNet	Multi-source Cohorts	216 human NSCLC patients	6-class cell-type nuclear features and Tissue classifier random forest	Characterize and quantify 6 distinct cell types in the NSCLC TME to identify TME heterogeneity and its prognostic value directly from standard H&E slides	DuCote et al., 2023	(30)
PET/CT	DL	CNN + Bidirectional RNN	Public database	211 subjects	10-fold repeated stratified cross-validation	Non-invasive and automatic prediction of major NSCLC histological subtypes from pre-treatment PET/CT images	Moitra et al., 2020	(33)
MRI	ML	CNN	Double-center database	151 patients	Xgboost	identification of brain metastases, AUC 0.80	Deng et al., 2023	(34)
CT	Radiomics and ML/DL	LightGBM and 3D-CNN	Public database	422 patients	PyRadiomics	Non-invasive, preoperative prediction of major NSCLC histological subtypes using CT-based radiomic features	Panchawagh et al., 2025	(35)
CT	DL	MMDL	Single-center	185subjects	Multi-channel raw ROI inputs	Simultaneous and non-invasive prediction of both EGFR and KRAS mutation statuses in NSCLC patients using preoperative CT images, AUC = 0.8083, Accuracy = 78.38% (EGFR), AUC = 0.8250, Accuracy = 83.78% (KRAS)	Dong et al., 2021	(38)
CT	Radiomics + DL + Clinical	CNN + LightGBM	Multi-center Cohorts	Internal Train: 885 patients External Test: 395 patients	3D ROI Segmentation + CNN Bottleneck + LASSO Cox/Logistic	Develop and externally validate an integrated deep learning-radiomics model for noninvasive detection of EGFR mutations in NSCLC. AUC = 0.842(Internal Cohort). AUC = 0.793(External Validation Cohort)	Kim et al., 2024	(40)
Brain MRI	DL	ResNet50	Single-center	87 patients with 137 brain metastases	2D ROI Bounding Box	Non-invasive detection of EGFR mutation status directly from Brain MRI of NSCLC brain metastases, overcoming tissue molecular discordance, Accuracy: 81.2%, Sensitivity: 81.8%	Haim et al., 2022	(41)
PET/CT	DL	EfficientNet-V2	Yunnan Cancer Hospital	150 patients	3D ROI Segmentation	Predicting the mutation status of EGFR gene based on an integrated PET/CT image of NSCLC. Accuracy rate in the validation set: 81.92%	Xiao et al., 2023	(42)
Brain MRI	DL	ResNet50-SGE	Multi-center Cohorts	284 patients with 432 brain metastases	3D ROI Segmentation + SGE Attention Map	Predict EGFR mutation status and its key molecular subtypes (19Del vs. 21L858R) directly from Brain MRI. EGFR Status: External Test AUC = 0.819, Accuracy = 82.4%	Cao et al., 2024	(43)

### 2.1 AI-based histopathological diagnosis

In the precision diagnosis and treatment workflow of NSCLC, histopathological diagnosis and molecular phenotyping serve as the cornerstones for formulating individualized treatment plans (18, 19). Recently, AI-assisted diagnostic techniques have made considerable progress in the field of histopathology. A substantial body of evidence-based medical data indicates that the integration of AI-related technologies has not only substantially improved the accuracy and efficiency of pathological diagnosis, but also demonstrated significant clinical value in shortening turnaround times and reducing healthcare costs (20, 21).

At the level of routine morphological screening and subtype classification, AI models have significantly increased the decision-making efficiency of pathologists by deeply mining geometric topological and textural features from digital slides (22). A study by Masot et al. compared the performance of three CNN-based classification pathways in the recognition of lung tissue pathological images: a “color image + three-category classification” model, a “grayscale image + three-category classification” model, and a purely “benign vs. malignant binary classification” model (23). The results revealed that the color image pathway, which preserves rich color information, achieved significantly better diagnostic metrics than the grayscale image pathway did; although the binary classification model did not further distinguish tumor subtypes, it offered a substantial time-cost advantage in large-scale clinical screening. Notably, the team innovatively introduced heatmap visualization and confidence percentage scores into the pathological reports, thereby achieving a dual improvement in the reading efficiency and subjective diagnostic accuracy of pathology experts.

However, conventional pathological diagnosis is still limited by the cumbersome preparation of tissue sections. To address this, Zang et al. overcame this limitation by employing deep learning technology combined with label-free autofluorescence, achieving ultrarapid and accurate pathological subtyping through the intelligent recognition of thyroid transcription factor-1 (TTF-1) expression specific to lung adenocarcinoma (LUAD) and p40 protein expression specific to squamous cell carcinoma (LUSC) (24). This approach eliminates the time-consuming conventional immunohistochemistry (IHC) processing and staining steps.

Although genomic alterations such as EGFR mutations and ALK/ROS1 gene fusions play a central role in the targeted therapy of NSCLC, in the real world, a considerable proportion of advanced-stage patients miss the optimal window for molecular testing because of the high cost of next-generation sequencing (NGS), long turnaround times (TATs), and high equipment requirements for primary hospitals (25). To address this clinical challenge, another group of researchers designed a computer vision deep learning method capable of predicting the gene fusion status of ALK and ROS1 with high accuracy by directly examining routine hematoxylin and eosin (H&E)-stained whole-slide images (WSIs) (26). Similarly, Rolfo et al. developed the CanvOI 1.1 model, which also achieves the intelligent identification of core driver gene alterations, including those of EGFR, ALK, BRAF, and MET, on the basis solely of routine H&E slides (27). This “image-to-genome” AI prediction paradigm holds promise as a highly cost-effective “virtual NGS” screening tool, enabling patients in economically underdeveloped regions to receive appropriate first-line targeted therapy in a timely and low-cost manner.

Moreover, AI has provided pathological diagnosis with a deeper dimension of prognostic assessment. As NSCLC treatment has fully entered the immunotherapy era, the assessment of spatial heterogeneity within the tumor microenvironment (TME) has become increasingly important (28, 29). A recent study employed the HALO® AI nuclear phenotyper to perform high-throughput intelligent recognition, spatial quantification, and benchmark comparisons of cell type distribution on H&E-stained sections (30). This technology can transform complex immune cell infiltration, stromal components, and spatial interactions of tumor nests into digitized continuous variables. In the future, the development of AI in pathological diagnostics is poised to realize virtual immunohistochemistry in the context of rapid frozen sections, thereby accelerating the advancement of point-of-care surgery while further advancing the development of spatial omics by quantifying the four-dimensional spatial architecture of the tumor environment.

### 2.2 AI-based imaging radiomics diagnosis

Needle biopsy, as the “gold standard” for histopathological examination, has long held a dominant position in cancer diagnosis. However, owing to its inherent invasiveness, heterogeneous sampling bias, and high risk of puncture at certain anatomical sites, not all patients can tolerate this procedure clinically (31). Fortunately, the convergence of AI and medical imaging has opened a new avenue to address this clinical challenge. Through noninvasive and reproducible quantitative radiomics, AI is capable of mining spatial textural heterogeneity that is imperceptible to the human eye, thereby enabling precise stratification of NSCLC at the macroscopic level for subtype prediction and driver gene analysis (32).

In this context, the synergy between multimodal imaging and advanced algorithms has demonstrated significant advantages. Moitra et al. proposed an innovative integrated architecture that organically combines Clubbing CNN with Bidirectional Recurrent Neural Networks (Clubbing CNN-BiRNN) to analyze PET/CT images from 211 subjects (33). This multimodal ensemble model significantly outperformed any single-network architecture in predicting NSCLC subtypes. Given that the brain is the most common target organ for NSCLC metastasis, another study used a different approach and developed a radiomics model based on the brain MR images of 151 patients. It trained a 3D-CNN via transfer learning to reversely identify the histological subtype of the primary NSCLC and incorporated the SHAP algorithm to provide visualized causal explanations for the extracted features, substantially enhancing the credibility of the model's decision-making (34). Moreover, the fusion of traditional handcrafted features and deep features has further established the robustness of noninvasive diagnosis. A group of researchers combined hand-crafted quantitative radiomics with deep learning and machine learning, successfully achieving precise quantitative determination of NSCLC histological subtypes through routine preoperative CT scans (35). These studies confirm that the noninvasive paradigm of AI plus radiomics can provide high-value histological references for preoperative treatment decision-making without the need to rely on invasive examinations. However, the reported performance should be interpreted cautiously: the cohorts were modest in size, the imaging modalities and endpoints differed, and not all models underwent independent external validation, limiting direct comparison and transportability.

Gene mutation status serves as the absolute gold standard for formulating targeted therapy regimens in advanced NSCLC, making the construction of “virtual gene testing” models a research focus in academia in recent years (36). Li et al. developed a multimodal diagnostic model, LG-MutaNet, which innovatively performs deep matrix fusion of patients' CT image features with key clinical covariates (age, sex, and smoking status), achieving high-precision joint prediction of EGFR and KRAS mutation status (37). To further increase the depth of feature extraction, Dong et al. constructed a multichannel and multitask deep learning (MMDL) model, which directly mines hidden phenotypes related to genetic heterogeneity from CT images through a synergistic learning mechanism (38, 39).

In terms of large-sample validation and application in specific clinical scenarios, the chain of clinical evidence is becoming increasingly complete. One group of researchers trained a CNN model using a large-scale dataset of pretreatment CT images from 1,280 NSCLC patients, extracted up to 512 high-order image features, and integrated these features with clinical data to accurately predict EGFR mutations while also evaluating the generalizability of the model through external multicenter data, confirming the robustness of this technique across different devices (40). In the more challenging scenario of brain metastasis (BM), Haim et al. retrospectively analyzed clinical, radiological, and molecular pathological data spanning a decade from patients who underwent resection of lung cancer brain metastases. They overcame the training bottleneck of small-sample brain metastasis data by employing data augmentation and transfer learning techniques, successfully constructing an AI model capable of accurately predicting the EGFR mutation status of brain metastases (41). Additionally, Xiao et al. and Cao et al., leveraging the EfficientNet-V2 architecture and a multiscale feature fusion network (MSF-Net), respectively, successfully predicted EGFR mutation status in primary lesions and brain metastases of primary NSCLC using PET/CT and MR images, further enriching the technical landscape of multimodal imaging genomics (42, 43). Nevertheless, much of this evidence remains retrospective; performance across centers may reflect differences in acquisition protocols and case mix as well as model robustness.

In the future, the advancement of AI in radiomics may help economically underdeveloped regions construct screening filters using routine CT scans to identify patients with gene mutations, thereby facilitating further treatment planning. Moreover, radiomics can capture dynamic feature evolution through regular follow-up examinations. During its development, it is crucial to focus on standardizing imaging protocols and improving the sensitivity of key metrics to enhance model credibility.

## 3 Application of AI in the treatment of NSCLC

**Figure 2:**
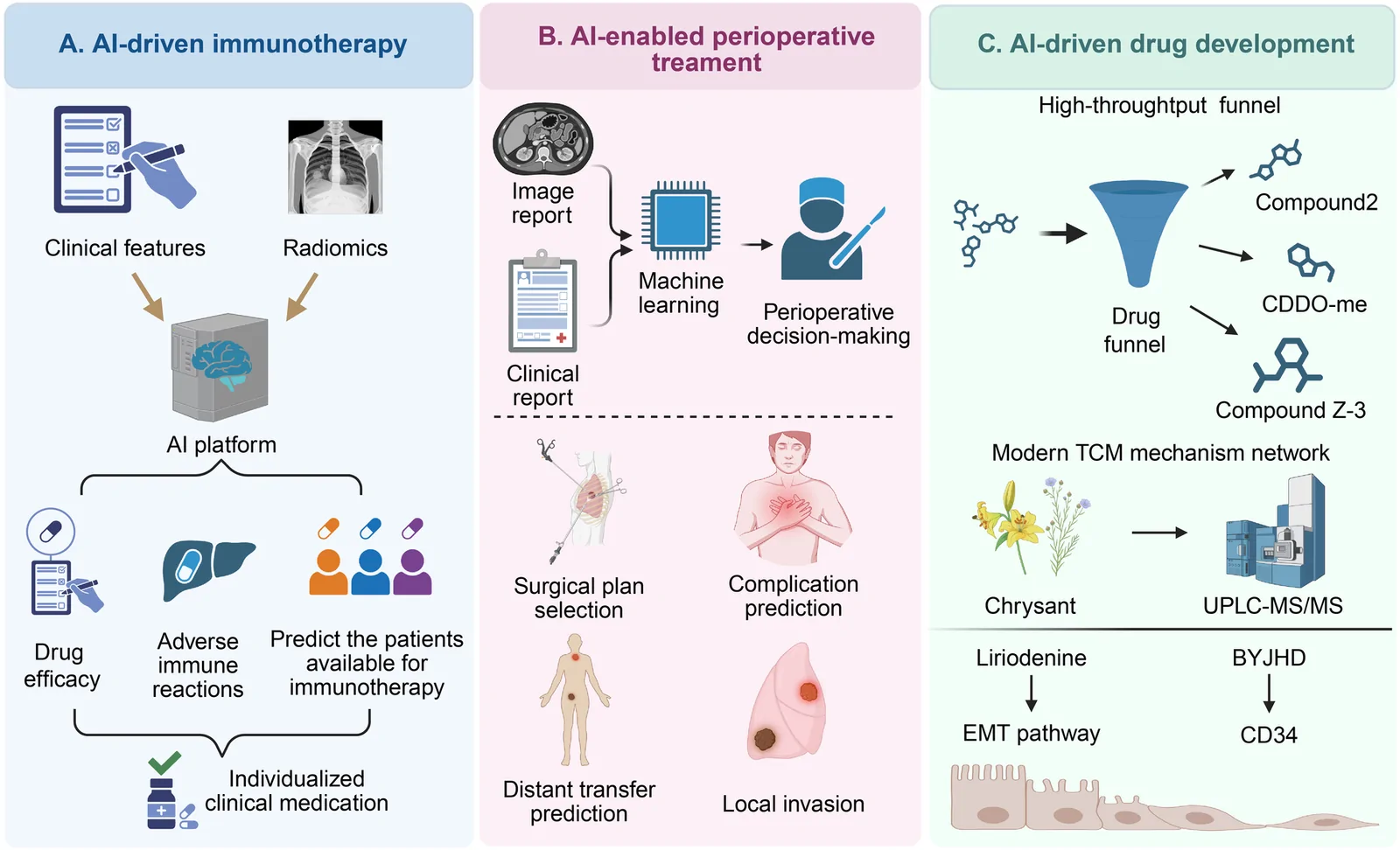
Application of AI in the treatment of NSCLC. **(A)** AI-drive immunotherapy. **(B)** AI-enabled perioperative treatment. **(C)** AI-driven drug development.

AI is fundamentally transforming the therapeutic landscape of NSCLC by facilitating highly personalized interventions. In this section, we review the integration of AI across multiple therapeutic domains. Specifically, we summarize its critical applications in predicting responses to immunotherapy and targeted therapies, optimizing perioperative surgical decision-making, and accelerating novel drug discovery and compound screening (Table 2) (Figure 2).

**Table 2:** Application of AI in the treatment of NSCLC.

Imaging modality	Model	Classifier (ML)	Design/source	Sample/valid	Feature/valid	Purpose and results	Author and year	Reference
Electronic Health Records	LLM + NLP	Ensemble of Large Language Models	Single-center Big Data Registry	2,154 NSCLC patients	Pre-defined rule-based prompt-like criteria	Using validated LLM-based AI to automatically extract RWD from unstructured text in EHRs to analyze EGFR mutation prevalence and survival outcomes in Canadian NSCLC cohorts. 136 (22%) had common sensitizing EGFR mutations, and 8 had exon 20 insertions	Moulson et al., 2024	(48)
—	ML	NN, Random Forest, Gradient Boosting, XGBoost, Logistic Regression	Single-center	397 patients	Baseline filtering + SHAP	Predict first-line ICI treatment response and the occurrence of irAEs simultaneously in NSCLC patients using readily available clinical data. AUC = 0.84, Recall = 84.06%, F1-score = 0.8007	Liu et al., 2025	(51)
CT	Multimodal ML + LLM	Random Forest + Bayesian Graph + LLM Fine-tuning	Public Database	187 patients	Tumoral and Peritumoral ROIs + Clinicopathological (PD-L1, TMB) Data	Develop a clinically explainable multimodal ML and LLM-integrated model to predict immunotherapy response in NSCLC from tumor/peritumoral radiomics and clinical biomarkers. AUC = 0.83	Mitra et al., 2025	(55)
Digital Pathology	AI-powered Spatial TME Analyzer	Deep Learning-based Semantic Segmentation & Cell Detection	Multi-cohort Study	143 patients	Spatial TME Metrics	Characterize the spatial remodeling of TME constituents after EGFR-TKI resistance and predict the clinical efficacy of subsequent immune-checkpoint inhibitor (ICI)-based therapies	Bang et al., 2025	(57)
PET/CT	Hybrid DL Network	CNN-ViT + Logistic Regression/SVM	Single-center Retrospective Registry	167 patients	3D Volumetric ROI + Automated Attention Token Selection	Develop and validate a hybrid CNN-ViT deep learning model using preoperative PET/CT to predict distant metastasis (DM) in NSCLC. AUC:0.88(training cohort), AUC：0.82(independent validation cohort)	Hu et al., 2026	(59)
PET/CT	Deep Learning Radiomics DLR Framework	ResNet-50 + LR Ensemble	Multi-center Study	186 patients	3D Lymph Node ROI + LASSO Regression	Develop and validate a PET/CT-based DLR model to non-invasively predict mediastinal lymph node metastasis in patients with NSCLC	Duan et al., 2026	(61)
High-Resolution CT	Integrated RDL Model	ResNet-50 + LightGBM	Multi-center Cohorts	421 patients	3D Volumetric ROI + Max-Relevance mRMR + LASSO	Develop and validate an integrated RDL clinical nomogram to non-invasively predict the presence of STAS in stage T1 NSCLC patients preoperatively. AUC:0.841(External Validation Cohort 1), AUC:0.814(External Validation Cohort 2)	Xu et al., 2025	(62)
High-Resolution CT	Interpretable ML Model	XGBoost + SHAP	Single-center Retrospective Registry	584 patients	Demographic Factors + Visual CT Semantics + Multivariable Logistic Regression	Develop and validate an explainable machine learning model based on preoperative clinical and semantic CT features to predict the presence of STAS in resectable NSCLC patients. AUC:0.841(training cohort), AUC:0.814(independent validation cohort)	Yang et al., 2026	(64)
—	FCNN	FCNN + K-Layer Cross-Validation Ensemble	Single-center Retrospective Registry	271 patients	Multivariable Preoperative Phenotyping	Develop and validate an artificial intelligence-assisted, non-invasive risk prediction model using FCNN to preoperatively forecast PPCs in NSCLC surgery patients. AUC:90.4%(test)	Özçıbık Işık et al., 2026	(65)
Protein x-ray Crystallography	AI-based + Docking-based VS Framework	DL for ADMET + Glide Molecular Docking	Public Biomolecular Database	6 c-MET Protein Structures + Large-scale Chemical Compound Library	Hydrogen-bond Assignment + Energy Minimization + Multi-descriptor ADMET Filtering	Discover novel and potent small-molecule c-MET inhibitors for NSCLC treatment by integrating AI-based predictive screening and molecular docking	Shen et al., 2026	(70)
Molecular Dynamic Simulations and *in vivo* Bioluminescence Imaging	Integrated Bayesian Inference Machine Learning Framework	Laplacian-modified Naïve Bayesian Classifier + Molecular Docking	Public Database	70,413 Molecules Screened	FCFP_6 and ECFP_6 Molecular Fingerprints + Structural Descriptors	Develop an integrated machine learning approach to screen large-scale chemical libraries for discovering novel, potent, and selective inhibitors targeting EGFR T790M-mutant NSCLC to overcome acquired TKI resistance. ROC-AUC: 0.908	Zhou et al., 2024	(71)
Molecular Docking and Molecular Dynamics Simulations	Fingerprint-enhanced Graph Attention Convolutional Network	GAT + GCN + Multilayer Perceptron Regressor	Public Bioassay Database + Commercial Virtual Library	5,479 Training Compounds	Combined Node-Edge Graph Attributes + MACCS and PubChem Fingerprints	Develop a novel multimodal deep learning model (FnGATGCN) to accurately predict VEGFR2 inhibitor activity and efficiently screen chemical libraries to discover new targeting drug candidates for NSCLC treatment	Wang et al., 2024	(72)
Molecular Docking and Molecular Dynamics Simulations	Network Pharmacology + Machine Learning Screening Pipeline	RF + LASSO Regression Algorithm	Public Biomolecular Databases + Experimental Validation	65 Overlapping Targets	Target-Disease Gini Importance + Penalty Coefficient Tuning	Identify the anti-NSCLC targets of liriodenine using integrated Random Forest and LASSO algorithms	Yang et al., 2026	(74)
Molecular Docking and *in vivo* Tumor Xenograph Imaging	Multi-ML Integrated Network Pharmacology Framework	LASSO + SVM-RFE + RF Ensemble Classifier	TCMSP + Serum Pharmacochemistry + TCGA NSCLC Dataset	38 Bioactive Compounds	LASSO + SVM-RFE + RF	Decypher the anti-angiogenic targets of Baiying Juhua Decoction in NSCLC using an ensemble machine learning pipeline (LASSO/SVM-RFE/RF)	Meng et al., 2026	(75)
Pathological WSI and Structural Target Cryptography	AI-Powered Digital Pathology Segmentation + Integrated Machine Learning	XGBoost + RF + LASSO + Deep Radiopathomics	TCGA-LUAD/LUSC Dataset + GEO + HERB and TCMSP Databases	16 Bioactive Monomers	Multivariable Weighting + Pathological Tile Feature Extraction	Screen anti-NSCLC targets of Lonicerae Japonicae Flos and evaluate immune microenvironment remodeling via multi-ML and AI digital pathology	Liu et al., 2025	(76)

### 3.1 AI in immunotherapy and targeted therapy

Targeted therapies and immunotherapies, including immune checkpoint inhibitors (ICIs), have substantially changed the treatment landscape of NSCLC (44). Multiple studies have demonstrated that AI models can accurately predict targeted therapy and immunotherapy responses as well as clinical benefits in NSCLC patients through the integration of multidimensional data (45, 46).

First, in the extraction of clinical real-world data and prediction of molecular subtypes, AI has laid a precise foundation for immunotherapy and targeted therapy (47). With the use of the validated DARWEN™ AI platform, real-world data from NSCLC patients can be automatically extracted from EHRs. In a study involving 613 advanced-stage cases, AI accurately identified EGFR mutation subtypes and treatment pathways and revealed that the one-year survival rate of patients with sensitizing mutations after developing resistance to osimertinib was only 20%–35%, providing an important reference for subsequent immunotherapy in patients with rare mutations or drug resistances (48, 49).

Second, in the precise prediction of immunotherapy response and immune-related adverse events (irAEs), machine learning algorithms have shown robust quantitative assessment capabilities (50). A retrospective study of 397 patients treated with ICIs compared five machine learning models. The results revealed that the neural network model performed best in predicting immunotherapy efficacy (test set AUC 0.84), whereas the random forest model led to the prediction of irAEs (test set AUC 0.82) (51, 52). Feature importance analysis further revealed red blood cell count, metastatic site, and sex as key predictors, providing a novel model for the individualized selection of ICI drugs. However, a limitation of this model is that its key predictors are not recognized immune biomarkers but rather rely on peripheral blood parameters and demographic characteristics, which diminishes its clinical persuasiveness (53).

Finally, in multimodal fusion innovation and dynamic monitoring of the tumor microenvironment (TME), AI is advancing toward deeper clinical interpretability and spatial landscape characterization (54). One study constructed a fusion architecture of machine learning (ML) and a large language model (LLM). By integrating clinicopathological indicators and intratumoral/peritumoral CT radiomic features, the model achieved an area under the curve (AUC) of 0.83 in an independent test set. Its core innovation lies in using ML to construct a knowledge graph and fine-tuning the LLM to transform the complex graph structure into clinically readable sentences, thereby significantly enhancing the model's human-computer interaction and interpretability (55, 56). At the microscopic level, an AI-powered whole-slide image analyzer was used to assess dynamic TME changes after EGFR-TKI resistance. This study confirmed that EGFR-TKIs can reshape the tumor immune landscape and that tumor-infiltrating lymphocytes (TILs) and endothelial cells (ECs) within the cancer area (CA) can serve as important predictive biomarkers for subsequent combined immunotherapy, offering a new direction for TME-based immunotherapy design (57). However, this study still essentially relies on invasive pathological sections and provides only a static single-time-point assessment, without achieving longitudinal dynamic inference through noninvasive means during the course of treatment.

Despite these limitations, the aforementioned studies collectively demonstrate that AI can integrate macroscopic clinical features with microscopic immune mechanisms, thereby enabling precise identification of the optimal immunotherapy window and supporting clinicians in developing personalized treatment strategies.

### 3.2 AI in perioperative treatment

In the evolving landscape of surgical diagnosis and treatment for NSCLC, AI is being integrated into the entire perioperative decision-making process at increasing depth, enabling multidimensional support from the assessment of lymphatic and distant metastasis to the prevention of surgical complications (58). First, in the context of metastasis risk assessment and surgical decision-making, accurate prediction of metastatic pathways is crucial for formulating immunotherapy combinations or surgical strategies. For distant metastasis, a hybrid deep learning model combining CNN and Vision Transformer (ViT) architectures leverages the advantage of ViT in capturing long-range dependencies; its PET/CT multimodal fusion model achieved an AUC of 0.882 in predicting distant metastasis, substantially outperforming the conventional ResNet5 (59, 60).

With respect to locoregional metastasis and invasion, for differentiating benign from malignant mediastinal lymph node metastasis, an artificial neural network (ANN) and extratrees (ET) model incorporating multiple deep learning features exhibited the best performance (61); for spread through air spaces (STAS), a common mode of dissemination, a classification model integrating deep features and multiregional radiomics can provide a reliable basis for surgical planning in patients with T1-stage disease (62, 63). Moreover, in a study analyzing more than 500 surgical patients, the XGBoost model emerged as the optimal algorithm for predicting STAS status, with a test set AUC of 0.764, and SHAP analysis was employed to endow the model with clinical interpretability, successfully identifying key features such as nodule type, lobulation sign, and smoking history (64).

To further ensure perioperative safety, a fully connected deep neural network-based model integrating multidimensional parameters, including clinical, laboratory, and pulmonary function data, was used to efficiently predict postoperative pulmonary complications, with an accuracy of 90.4% and an AUC of 0.84 (65).

In summary, multimodel fusion approaches can outperform single-model approaches, while noninvasive assessments based on imaging and clinical data may support better perioperative risk stratification. However, the aforementioned studies are predominantly retrospective analyses, which lead to limited clinical translation capability, and they suffer from an overreliance on high-quality data. Future work should test whether through further advancement toward industrialization, AI will become an indispensable assistant to surgeons.

### 3.3 AI in drug discovery and development

Currently, through high-throughput screening, multimodal molecular characterization, and complex machine learning, AI is addressing longstanding bottlenecks in lengthy development cycles and low success rates in traditional drug discovery, playing a central role in targeted drug development, the discovery of resistance-focused lead compounds, and the mechanistic elucidation of modernized traditional Chinese medicine (TCM) (66–68). First, in the development of anti-resistance and novel small-molecule targeted drugs, AI has demonstrated a powerful capacity for exhaustive chemical space searching and rational design (69). By targeting c-MET, a key oncogenic driver in NSCLC, AI successfully identified and validated novel small-molecule c-MET inhibitors from large-scale compound libraries, among which “Compound 2” not only potently inhibited NSCLC cell proliferation but also possessed favorable ADMET drug-like properties (70). To address the challenge of overcoming classical targeted therapy resistance driven by the EGFR T790M mutation, researchers have employed a Bayesian inference machine learning method to efficiently screen a massive database of more than 70,000 molecules and discovered a novel selective inhibitor, CDDO-Me; molecular dynamics simulations, CETSA, and both *in vitro* and *in vivo* experiments have collectively confirmed that this compound selectively binds to mutant EGFR and blocks the PI3K-Akt-mTOR signaling axis (71). Moreover, in the development of agents targeting angiogenesis, a novel multimodal FnGATGCN model that combines molecular graph and fingerprint features to predict bioactivity successfully identified 11 VEGFR2-stabilizing molecules after screening the ZINC database combined with dynamic simulations, among which “Compound Z-3” exhibited potent and low-toxicity characteristics against both VEGFR and A549 cells (72).

Second, in the modernization of TCM and the discovery of candidate drugs, AI provides quantitative tools for deciphering the multicomponent, multitarget mechanisms of herbal formulas and natural products (73). A Chinese research team used random forest (RF) and least absolute shrinkage and selection operator (LASSO) regression algorithms to precisely screen public databases and identified the natural product liriodenine as a core therapeutic target agent in NSCLC, revealing that it exerts antitumor effects by inhibiting the TGF-*β*-mediated EMT pathway (74). Similarly, a DL model combined with the LASSO algorithm was used to systematically analyze the anti-NSCLC mechanisms of Baiying Juhua Decoction (BYJHD), and 38 active compounds and 54 key therapeutic targets were efficiently identified, ultimately confirming its antiangiogenic activity through inhibition of the ACVRL1/Smad/ID-1 signaling pathway and downregulation of CD34 expression (75). In another study, UPLC‒MS/MS technology was integrated with network pharmacology and multiple machine learning algorithms to systematically elucidate the molecular mechanisms of honeysuckle (*Lonicera japonica*) as an adjuvant therapy for NSCLC, screening PECAM1 and SPP1 as core signature targets closely associated with tumor immune evasion; *in vitro* experiments confirmed that honeysuckle significantly inhibited A549 cell proliferation and migration while upregulating the expression of the above proteins (76).

These studies indicate that AI acts as a funnel to screen existing compounds in libraries while simultaneously identifying TCM formulas and natural products that were previously difficult to quantify precisely. In the future, AI may not only repurpose existing drugs but also leverage generative AI to design entirely novel dual-target small molecules with new scaffolds.

However, these studies also have several limitations. For instance, only CDDO-Me was explicitly reported to have undergone *in vivo* experiments, while the others were confined to *in vitro* proliferation and migration assays. Moreover, after oral administration of TCM formulas, the substances that actually enter the human body are often metabolites produced by the gut microbiota rather than the original compounds, which may cause the models to overlook *in vivo* metabolic processes. Currently, the main challenge for AI in NSCLC drug discovery is the disconnect between high-throughput virtual screening capability and low-throughput biological validation throughput. With further clinical validation, AI models are expected to find even broader applications in drug discovery and development.

## 4 Application of AI in the prognostic evaluation of NSCLC

**Figure 3:**
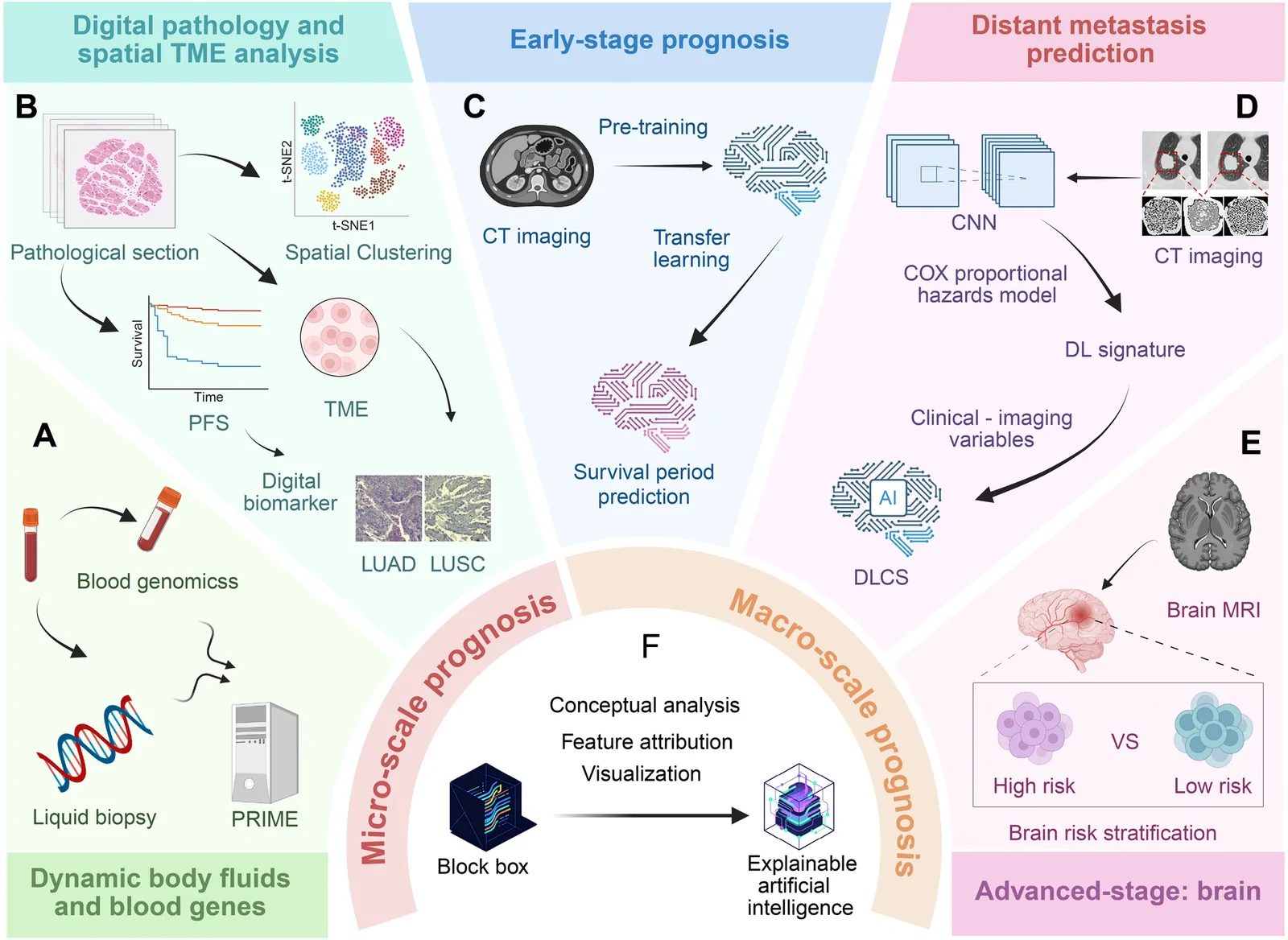
Application of AI in the prognostic evaluation of NSCLC. **(A)** AI application for prognosis assessment in dynamic body fluid and blood genomes. **(B)** AI Application in prognostic assessment of digital pathology and spatial TME analysis. **(C)** AI application in early diagnosis. **(D)** The prognostic application of AI in distant metastasis. **(E)** AI application in the prognosis of brain metastases. **(F)** Machine learning explainability Artificial intelligence.

Accurate prognostic assessment is crucial for optimizing survival outcomes and personalizing post-treatment management in NSCLC. In this section, we summarize the prognostic applications of artificial intelligence across multiple dimensions (Figure 3). We review the use of microscopic spatial omics in digital pathology and macroscopic imaging radiomics for predicting recurrence and metastasis (Table 3). We also highlight the integration of multimodal data and explainable AI to enhance clinical transparency and decision support.

**Table 3:** Application of AI in the prognosis of NSCLC.

Imaging modality	Model	Classifier (ML)	Design/source	Sample/valid	Feature/valid	Purpose and results	Author and year	Reference
Liquid Biopsy + Multi-omics	PRIME	Neural Network Optimized Classifier + SHAP	6 Global Multicenter Cohorts + TCGA Baseline	781 Longitudinal Blood Samples from 493 Patients	Integrated Blood-Clinical Multimodal Vector	To develop an interpretable AI model (PRIME) fusing longitudinal ctDNA-MRD and blood genomics for post-treatment NSCLC progression risk stratification	Wang et al., 2025	(78)
Digital Pathology H&E + Imaging Mass Cytometry	Cross-scale Radiopathomics & Hi-plex Spatial Topology Framework	AI-driven Semantic Segmentation + Spatial Clustering	Institution NSCLC Biobank (158 Specimen Cohort) + 29-antibody Hi-plex Panel	158 Digital HE Whole Slides	Architectural Spatial Clustering Index + 29-plex Single-cell Phenotypic Markers	To characterize the non-small cell lung cancer tumor microenvironment by combining AI-powered histopathology and hyper-plex imaging mass cytometry	Rigamonti et al., 2025	(81)
Digital Pathology Whole Slide Images	Expert-in-the-Loop Integrated Multiclass Segmentation U-Net Framework	U-Net Deep Architecture + RF Pixel	Institutional Chest CT-Pathology Linked Cohort	76 Full WSIs	Multi-class Spatial Pixel Topology	To segment and characterize the non-small cell lung cancer tumor microenvironment across fibrotic and non-fibrotic lung backgrounds using an expert-in-the-loop U-Net model	Saqi et al., 2024	(83)
Digital Pathology WSI	AI-powered Patho-immunoscore Analyzer	Deep CNN	TCGA-LUAD + CPTAC-LUAD + ORIENT-11 Phase 3 Clinical Trial	1,333 WSIs	Spatial Lymphocyte Density + Tumor-Infiltrating Immune Topology Features	To develop an artificial intelligence-based immunoscore to predict clinical outcomes of chemoimmunotherapy in advanced non-squamous NSCLC patients	Liu et al., 2024	(84)
Digital Pathology WSI	Weakly Supervised Multi-scale Pathomics Pipeline	DenseNet121 + XGBoost Classifier Framework	Multicenter NSCLC Cohort	212 WSIs	Pathomics Feature Bag-of-Words and TF-IDF Probability Distribution	To predict major pathological response in non-small cell lung cancer post-neoadjuvant chemoimmunotherapy using weakly supervised whole slide image deep learning	Han et al., 2024	(86)
Preoperative Chest CT	DL Prognostic Model	3D CNN	SNUH Institutional Database + KCCH Biobank	1,073 Patients	Deep Radiomics Spatial Features	To evaluate a CT-based deep learning prognostic model for selecting segmentectomy vs. lobectomy candidates in early-stage NSCLC	Na et al., 2024	(88)
Multimodal Longitudinal CT	Multimodal Deep Learning Biomarker Framework	3D-ResNet18 + CBAM Convolutional Neural Network	5 Multicenter Chinese Institutions + 1 Prospective Clinical Trial	358 Patients	Spatiotemporal Volume Tensors	To develop a multimodal CT deep learning biomarker for predicting pathological complete response in NSCLC following neoadjuvant immunochemotherapy	Ye et al., 2024	(90)
Preoperative Multimodal Chest CT	Deep Learning Clinical-Imaging Signature Framework	3D-CNN	Two Affiliated Academic Medical Center Databases	1,547 Patients	3D Tumor-Volume DL Signatures + Multiparametric Clinical-Imaging Risk Factors	To establish a preoperative CT-based deep learning signature to predict bone metastasis-free survival in non-small cell lung cancer	Guo et al., 2024	(91)
Preoperative Low-Dose Computed Tomography	NAS-Driven DL and Radiomics Cascaded Biomarker	NAS Auto-CNN + Least Absolute Shrinkage and Selection Operator	Two Major Academic Medical Centers	381 Retrospective	NAS-Automated Hidden Deep Features + Shape and Intratumoral Voxel Radiomics Maps	To develop an automated low-dose CT-based deep learning and radiomics model for predicting distal metastasis in non-small cell lung cancer	Song et al., 2024	(92)
T1 and T2 MRI	Radiomics-Transcriptomics Integrated Prognostic Framework	Bidirectional Stepwise Logistic Regression	/	Xiangya Hospital Institutional Database + Matched Brain Metastasis Tissue RNA-seq292 Multimodal MRI Samples	PyRadiomics-Derived 3D Texture Tokens + Microenvironmental Immune Expression Profile	To develop an interpretable radiomic model for predicting survival in non-small cell lung cancer patients with brain metastases	/	(94)
Pretreatment Chest CT	Deep Learning-Driven Segmentation and Radiomics Ensemble Model	3D Res-UNet + Extra Trees	Two Major Comprehensive Cancer Centers	602 Patients	PyRadiomics 3D Shape + Intratumoral Heterogeneity	To establish an ensemble learning model based on CT radiomics and deep learning segmentation to predict brain metastasis in advanced NSCLC	Gong et al., 2024	(95)
Multimodal Brain Imaging	Radiomics-Based ML Meta-Analysis Framework	Random Forest, SVM, LightGBM, and Logistic Regression Classifiers	Global Multi-database Pooling	3,517 Patients	Multi-parameter Image Radiomics	To evaluate the predictive performance of machine learning-based models for EGFR mutation status in NSCLC brain metastases	Hajikarimloo et al., 2025	(96)
Preoperative Multimodal Chest CT	Integrated DL Radiomics and Mediastinal Adipose Nomogram	CNN Feature Extractor + Multivariate Logistic Regression	Institutional Thoracic Surgery and Radiology Databases	585 Postoperative NSCLC Patients	Mediastinal Fat Area + CNN-derived DLBMS + Clinical TNM Stage and Pathology	To develop a predictive nomogram integrating mediastinal fat area and deep learning tumor characteristics for postoperative brain metastasis in NSCLC	Niu et al., 2025	(97)
PET/CT	DL	CNN	Multi-database	590 Patients	Combined risk stratification	To improving the predictive ability of TNM staging	Ju et al., 2024	(101)
Pre-treatment Clinical-Staging Metrics and Radiotherapeutic Dosimetric Parameters	Explainable DL and ML Survival Prediction Models	RSF and CPH	Large-scale Quality-assured Radiotherapy Institutional Database	711 Patients	Multiparametric Pre-treatment Covariables	To compare deep learning with machine learning to predict overall survival in non-small cell lung cancer patients receiving radiotherapy	Astley et al., 2024	(102)
Preoperative Multiparametric Clinical Metrics and Perisurgical Physiological Phenotypes	Machine Learning-Based Postoperative Frailty Predictive and Stratification Framework	Extreme Gradient Boosting benchmarked against Random Forest, SVM, and LR	Two Affiliated Academic Medical Center Databases	303 Postoperative NSCLC Patients	LASSO Regularization + SHAP-driven Multiparametric Clinical-Physiological Selection	To develop a machine learning model for predicting postoperative frailty in patients undergoing non-small cell lung cancer surgery	Su et al., 2025	(103)
3D CT Chest Radiomics + EHR Text Modality	ssuBERT-Driven Multimodal Integration Prognostic AI Framework	ssuBERT Integrated with Downstream Risk Classifier	Joint Real-World Hospital Cohort and Multi-Public Databases	452 Patients	ssGSEA Hypoxia Profiling + LASSO Feature Reduction + ssuBERT Token Recognition	To develop a multi-modal AI model predicting hypoxia status and prognosis in non-small cell lung cancer	Zhou et al., 2024	(104)

### 4.1 AI-based spatial omics prognostic assessment in digital pathology

AI technologies have demonstrated clinical potential in risk stratification and prognostic prediction for NSCLC, with a pronounced trend toward integrating single-modality data into multimodal and multiscale data fusion (77). At the multiomics microscopic level and in combination with liquid biopsy, to address the clinical challenge of postoperative recurrence risk assessment, the interpretable AI-assisted model PRIME, developed using multicenter global data, integrates multidimensional parameters, including liquid biopsy, blood genomics, and clinicopathological factors. After various algorithms were compared, the neural network model was established as superior, achieving an AUC of 0.82 in an independent validation set. SHAP-based interpretability analysis revealed that postoperative molecular residual disease (MRD), treatment modality, and preoperative ctDNA were the three core factors for predicting prognosis and revealed that specific gene mutations, such as those in KEAP1, STK11, and CDKN2A, could predict poor prognosis by inducing an immunosuppressive tumor microenvironment (TME) (78, 79). A major limitation of this study is the inherent heterogeneity in the sampling time points of dynamic liquid biopsy within a retrospective cohort, necessitating future prospective studies to standardize the sampling schedule.

In the realms of digital pathology omics and spatial microenvironment analysis, by integrating AI image analysis and high-plex imaging mass cytometry (IMC), researchers have been able to deeply dissect the microenvironmental ecology of NSCLC specimens (80). The AI approach successfully achieved precise identification of adenocarcinoma and squamous cell carcinoma cells and constructed a prognostic classifier based on spatial clustering features; IMC quantitatively detected 24 immune and structural markers and identified 11 macrophage clusters and the spatial distribution characteristics of T cells, confirming that intratumoral T-cell infiltration is significantly associated with better patient prognosis (81, 82). Moreover, a lightweight random forest algorithm-based CNN model developed using hematoxylin and eosin (H&E)-stained whole-slide images (WSIs) achieved high-accuracy tissue-level multiclass segmentation through an “expert-in-the-loop” (EITL) iterative procedure, although this inevitably introduces subjective annotation bias. Quantitative analysis confirmed that in a background of fibrosis, adenocarcinoma and squamous cell carcinoma exhibit significant TME heterogeneity in terms of the proportions of fibrosis, inflammation, blood vessels, and necrosis, demonstrating the clear feasibility of quantitative TME assessment based solely on H&E slides (83).

Moreover, an AI-driven pathological immunoscore analyzer applied to digital slides of lung adenocarcinoma demonstrated robust accuracy across multiple cohorts, including TCGA-LUAD, CPTAC-LUAD, and a large-scale randomized phase 3 clinical trial (ORIENT-11). The results confirmed that among patients who received chemotherapy combined with immunotherapy, the group with a high AI immunoscore had significantly superior median progression-free survival (PFS) than the group with a low AI immunoscore did, whereas this score was not different between the chemotherapy-alone group and the chemotherapy-only group, providing a noninvasive digital biomarker for immunochemotherapy in advanced nonsquamous NSCLC (84, 85). To predict the response to neoadjuvant treatment in patients with resectable NSCLC, a weakly supervised deep learning multiple-instance learning model (using a DenseNet121 architecture) was applied to pretreated H&E-stained slides, and multiscale analysis was performed at both the patch level and the slide level. A fusion nomogram constructed by integrating pathomic features and clinical data through the XGBoost algorithm was able to predict the major pathological response (MPR) in patients receiving neoadjuvant immunochemotherapy (NICT) with high accuracy (86).

These studies focus on the microscopic level. Through various algorithms, AI not only mines minimal residual disease but also achieves quantitative assessments of spatial clustering, the TME, and immunoscores from different tumors by analyzing existing slides, thereby predicting patient survival. Admittedly, the aforementioned studies are still constrained by *post hoc*, noncausal interpretations of the output, making it difficult to directly guide the exploration of microscopic pathophysiological mechanisms.

### 4.2 AI in prognostic applications of imaging radiomics and brain metastasis

At the macroscopic level of imaging radiomics and deep learning feature extraction, AI models based on CT, PET/CT, and MRI provide powerful noninvasive tools for predicting the risk of distant metastasis (87). For patients with clinical stage IA NSCLC, a deep learning prognostic model integrating preoperative CT images and clinical features, after pretraining and transfer learning on data from a large cohort of lobectomy patients, achieved an AUC of 0.86 for predicting 2-year freedom from recurrence (FFR) in an independent test set of segmentectomy patients. Its sensitivity was significantly superior to that of the conventional JCOG sublobar resection eligibility criteria, enabling more effective identification of patients at high risk of postoperative recurrence (88, 89). To predict the efficacy of neoadjuvant immunotherapy combined with chemotherapy, a deep learning model named LUNAI-fCT, developed using pretreatment noncontrast and contrast-enhanced multimodal CT images, achieved an AUC of 0.866 through feature fusion in an independent external test set, significantly outperforming models based on either noncontrast or contrast-enhanced images alone, and providing interpretable visualization of efficacy prediction through SHAP analysis and Grad-CAM heatmaps (90).

In the assessment of distant metastasis risk, a deep learning clinical imaging signature (DLCS) model, constructed by extracting deep learning features via 3D CNNs from preoperative CT and integrating clinical and imaging factors, accurately predicted bone metastasis-free survival (BMFS) after complete resection in NSCLC patients. In the dual-center validation sets, the C-index exceeded 0.80, and the model significantly stratified patients across various clinical stages into high- and low-risk groups for bone metastasis (91). Moreover, a deep learning fusion model adaptively developed using radiomics from low-dose CT (LDCT) and the NAS achieved an area under the curve (AUC) of 0.780 for predicting distant metastasis in multicenter external validation. Through radiomic voxel mapping and attention weight tracking, this model successfully enabled the quantitative detection of microscopic changes within tumor voxels, providing a digital basis for optimizing precise clinical intervention after lung cancer screening (92).

In particular, for brain metastasis (BM), the most common and serious complication of NSCLC, AI has achieved breakthroughs in risk prediction and mechanistic exploration (93). An interpretable prognostic model developed by integrating MRI radiomic features and RNA sequencing data achieved an AUC of 0.84 in the test set. Beyond risk stratification, immune infiltration analysis further confirmed that the interferon (IFN) pathway was significantly enriched and accompanied by CD8+ T-cell infiltration in the TME of the low-risk group (94). Another study integrated an automated segmentation technique using a 3D deep residual U-Net network with the XGBoost ensemble learning algorithm to deeply mine the imaging phenotype of the primary tumor from pretreatment chest CT images, accurately predicting the three-year risk of brain metastasis in advanced NSCLC patients, with an AUC of 0.85 in the external validation set. Survival analysis confirmed that the calculated risk score had a highly significant prognostic value for both brain metastasis-free survival (BMFS) and overall survival (OS) (95). In terms of genotype prediction, a systematic review and meta-analysis systematically evaluated the performance of AI in noninvasively predicting the EGFR mutation status in patients with brain metastases. The meta-analysis revealed that the pooled AUC of the optimal AI models was as high as 0.91, with a pooled accuracy of 0.82, and subgroup analysis indicated that traditional machine learning and deep learning demonstrated comparably robust performance in imaging feature mining (96).

Moreover, researchers have begun to focus on the influence of host metabolic status on metastasis by developing a composite nomogram model that integrates the mediastinal fat area (MFA) and a deep learning-based brain metastasis score (DLBMS), achieving an AUC as high as 0.947 in the test set. Both the integrated discrimination improvement (IDI) and the net reclassification improvement (NRI) confirmed that the introduction of MFA, a feature of the mediastinal adipose microenvironment, provided substantial incremental value in improving the predictive accuracy of the brain metastasis model (97). However, the measurement of MFA currently relies on manual or semiautomated quantitative assessment using ImageJ software and lacks a fully automated and standardized segmentation pipeline, which limits its utility in large-scale clinical workflows. Clinical imaging is the most convenient and noninvasive examination available during patient care. By focusing on macroscopic clinical images, AI can predict postoperative FFR in early-stage patients, as well as the risks of distant metastasis, bone metastasis, and brain metastasis in advanced-stage patients, while also encompassing certain applications of the AI at the primary care level.

### 4.3 Application of explainable AI in prognostic assessment

Finally, the integration of multimodal joint prediction and explainable AI (XAI) is emerging as a bridge to propel AI toward clinical decision support (98–100). A comprehensive risk stratification (CRS) model, constructed by combining CNN-based deep learning features from PET/CT with whole-body metabolic tumor volume (MTVwb), serves as an effective supplement to the conventional TNM staging system. Its fused nomogram achieved a C-index of 0.771 in the test set, significantly enhancing the prognostic assessment of overall survival (OS) and progression-free survival (PFS) (101). To address the linear limitations of traditional Cox proportional hazards (CPH) regression and the “black box” nature of machine learning, researchers introduced a survival-adapted LIME technique into a deep learning survival prediction model. This model demonstrated optimal performance for predicting OS in stage I-IV radiotherapy patients, with a C-index of 0.670 and an integrated Brier score of 0.121, and elucidated the prognostic contributions of covariates such as tumor stage, chemotherapy history, and mean esophageal radiation dose (102). However, SHAP, LIME, and Grad-CAM are *post hoc* explanation tools rather than evidence of causal reasoning. Their outputs can change with preprocessing choices, reference baselines, correlated features, or small image perturbations, and visually plausible heatmaps may highlight spurious dataset-specific signals. Clinical use therefore requires explanation-stability testing, clinician-concordance studies, and explicit human oversight; explanations should support, rather than replace, accountable clinical judgment. Because responsibility for an AI-assisted decision may be distributed across developers, institutions, and clinicians, prospective deployment should also define audit trails, escalation procedures, and medicolegal accountability.

AI models have even been extended to the systematic management of the entire disease course; for example, a model built using LASSO regression and the SMOTE algorithm effectively predicted frailty status three months after surgery and revealed its significant negative correlation with the quality of early recovery (103). With further bridging scales, a multimodal AI model was developed by defining microscopic hypoxia status through single-sample gene set enrichment analysis (ssGSEA) and integrating CT radiomic features with a natural language processing model, ssuBERT, specifically designed to analyze the semantic structural units of electronic health record (EHR) text. This model achieved a mean AUC of 0.8449 for predicting overall survival, significantly outperforming any single modality (104).

Although AI, through deep learning and quantitative mining of multicenter, multimodal, cross-scale data, has improved prognostic stratification for NSCLC patients in scenarios such as postoperative recurrence, distant and brain metastasis, neoadjuvant treatment response, and survival prediction, it is important to critically examine the common limitations inherent in the current experimental designs, including data completeness and acquisition costs, clinical confounding and bias, and insufficient algorithm generalizability. Reported metrics are not directly comparable across studies because cohorts, endpoints, preprocessing pipelines, and train-test split strategies differ substantially. The evidence is dominated by retrospective designs, selected populations, and internal validation, which can inflate apparent performance and obscure calibration failure, data leakage, subgroup inequity, and model drift. Even where external validation is reported, geographic and technical diversity is often limited, while incomplete disclosure of code, trained models, and preprocessing pipelines restricts reproducibility. Nevertheless, gradually advancing technologies are helping AI become a digital tool that can assist clinicians in formulating individualized precision treatment plans.

## 5 Perspectives and conclusion

With advances in AI technologies and the rapid iteration of large-scale models, AI has shown substantial promise in the precision diagnosis, treatment, and prognosis of NSCLC. In the diagnostic domain, AI leverages multimodal imaging, including CT, MRI, and PET/CT, to achieve noninvasive prediction of NSCLC subtypes, which can be further extended to the genetic level, holding the promise of substantially reducing testing turnaround times in the future (105). Moreover, the spatial sensitivity of AI enables early detection of metastasis, assisting clinicians in performing more precise staging. The application of AI in pathomics can facilitate faster intraoperative subtyping through digital pathology slides, providing additional markers for clinical diagnosis (106). In the therapeutic domain, AI integrates baseline multiomics data to construct highly efficient efficacy prediction models, thereby offering more robust predictive factors than traditional single indicators do. In terms of targeted therapy, immunotherapy, or drug development, AI can help identify more suitable patient populations or more effective drugs, optimize the selection of first-line treatment regimens and enable precision medicine (107). In the prognostic domain, by leveraging Kaplan‒Meier survival analysis and Cox proportional hazards models within deeply integrated algorithmic frameworks, AI can directly calculate individualized risk scores on the basis of preoperative imaging features or postoperative pathological spatial features, thereby achieving precise risk stratification (108, 109). Through longitudinal deep learning modeling of sequential follow-up images, AI can sensitively detect subtle drifts in high-order textural features before visible lesion enlargement on imaging, thereby providing early warning of drug resistance progression or recurrence of MRD.

Despite the remarkable achievements of AI research in NSCLC, the current technological system still faces many bottlenecks before achieving translation from “laboratory models” to “routine clinical diagnostic and therapeutic tools”. For instance, both imaging features and high-order nonlinear pathological features exhibit extremely high sensitivity to the conditions of underlying data acquisition and sample preparation (110, 111). The brands of medical imaging equipment, slice thickness, and reconstruction algorithms across different healthcare institutions, as well as the differences in staining batches, fixation times, and scanner color of pathological slides, can introduce serious result bias in real-world settings. Such data heterogeneity often leads to a precipitous decline in model performance during cross-center, cross-device external validation.

Currently, most high-performance deep learning models are still based on end-to-end “black box” architectures, and the high-dimensional abstract features they extract often lack clear molecular biological mechanisms or pathological anatomical correlations. The inability to provide clinicians with an intuitive causal chain has created a crisis of trust in medical practice, making it difficult for AI systems to be adopted as an independent basis in critical life-altering decisions. Moreover, certain AI models exhibit polarized diagnostic performance, particularly high specificity coupled with low sensitivity, in detecting occult lesions, potentially delaying timely diagnosis and compromising the therapeutic window.

In the future, AI research and applications in NSCLC will accelerate toward clinical translation and become deeply embedded in the management of the entire clinical disease course. Progress should be evaluated through staged prospective multicenter programs: cohort validation with prespecified endpoints, silent deployment to test workflow robustness, AI-assisted randomized or pragmatic trials to measure effects on decisions and patient outcomes, and post-implementation surveillance for calibration drift, subgroup performance, safety signals, and clinician override patterns. Such monitoring is essential because retrospective gains in AUC do not by themselves establish clinical utility or improved outcomes. Through the continuous assimilation of multidimensional time series data and online learning, dynamically adjusted digital predictive models can be tailored for each NSCLC patient, ultimately realizing true whole-lifecycle intelligent precision medicine.
